# Parental Supply of Alcohol in Childhood and Risky Drinking in Adolescence: Systematic Review and Meta-Analysis

**DOI:** 10.3390/ijerph14030287

**Published:** 2017-03-09

**Authors:** Sonia Sharmin, Kypros Kypri, Masuma Khanam, Monika Wadolowski, Raimondo Bruno, Richard P. Mattick

**Affiliations:** 1 School of Medicine and Public Health, University of Newcastle, Newcastle, NSW 2308, Australia; kypros.kypri@newcastle.edu.au; 2 School of Health Science, University of Tasmania, Hobart, TAS 7005, Australia; masuma.khanam@utas.edu.au; 3 Kirby Institute, University of NSW, Sydney, NSW 2052, Australia; mwadolowski@kirby.unsw.edu.au; 4 School of Psychology, University of Tasmania, Hobart, TAS 7005, Australia; Raimondo.Bruno@utas.edu.au; 5 National Drug and Alcohol Research Centre, University of NSW, Sydney, NSW 2052, Australia; R.Mattick@unsw.edu.au

**Keywords:** parental supply, alcohol, adolescent, risky drinking

## Abstract

Whether parental supply of alcohol affects the likelihood of later adolescent risky drinking remains unclear. We conducted a systematic review and meta-analysis to synthesize findings from longitudinal studies investigating this association. We searched eight electronic databases up to 10 September 2016 for relevant terms and included only original English language peer-reviewed journal articles with a prospective design. Two reviewers independently screened articles, extracted data and assessed risk of bias. Seven articles met inclusion criteria, six of which used analytic methods allowing for meta-analysis. In all seven studies, the follow-up period was ≥12 months and attrition ranged from 3% to 15%. Parental supply of alcohol was associated with subsequent risky drinking (odds ratio = 2.00, 95% confidence interval = 1.72, 2.32); however, there was substantial risk of confounding bias and publication bias. In all studies, measurement of exposure was problematic given the lack of distinction between parental supply of sips of alcohol versus whole drinks. In conclusion, parental supply of alcohol in childhood is associated with an increased likelihood of risky drinking later in adolescence. However, methodological limitations preclude a causal inference. More robust longitudinal studies are needed, with particular attention to distinguishing sips from whole drinks, measurement of likely confounders, and multivariable adjustment.

## 1. Introduction

Risky consumption of alcohol is a leading threat to adolescent health globally because of its role in the aetiology of intentional and unintentional injury, mental disorders, and sexually transmitted infection [1,2]. Risky drinking is defined as consumption of ≥5 drinks in a single episode at least monthly. The European School Survey Project on Alcohol and Other Drugs (ESPAD) reported that one in twelve adolescents at the age of 13 or below drank alcohol riskily in 2015 [3]. In the USA, 14% of 12–20 year-olds reported drinking ≥5 drinks on one or more occasions in the previous month, and this age group made 188,706 emergency room visits due to injury and other alcohol-related conditions in 2011 [4]. 

In addition to illegal sales, sources of alcohol for adolescent (i.e., under the legal age of purchase) drinking include parents, other relatives, and peers [5]. Parents may directly influence their children’s drinking by offering sips of alcoholic drinks at dinner or on special occasions, by supplying alcohol at supervised parties, or by permitting them to take alcohol to drink in unsupervised settings [6]. In Australia and the UK, where drinking per se is not illegal but where purchase is illegal under the age of 18 years, more than a third of adolescents report receiving alcohol from their parents [7,8]. Some research suggests that parents give their children alcohol to teach them how to drink responsibly and to prevent risky drinking with peers [9,10,11].

Research regarding the impact of parental supply of alcohol on adolescent risky drinking has produced conflicting results. A 2014 narrative review of the literature found that parental supply of alcohol was associated with heavy episodic drinking and higher risk of alcohol-related harm in 10 studies; but seven studies found it to be protective against such harm [6]. Some studies showed that parental supply was more prevalent in supervised than in unsupervised settings [12,13], with the latter being associated with a higher incidence of risky drinking among 13–17 year-olds [14]. In other studies parental supply was found to be associated with lower risk of hazardous drinking and related problems [15,16]. It is important to note that many of the studies included in the review were cross-sectional, such that the temporal relation between the hypothesized exposure and outcome could not be established. In addition, several studies did not adjust estimates for likely confounding variables (e.g., parent drinking [17,18,19]) so that estimates of association may be biased. Thus, the potential impact of parental supply of alcohol on adolescent risky drinking remains unclear.

There have been no reviews synthesizing longitudinal studies to examine associations between prospectively measured parental supply of alcohol and later adolescent risky drinking. We sought to critically examine longitudinal studies with prospective measurement of exposures, and to conduct a meta-analysis to determine whether parental supply of alcohol is associated with later risky drinking.

## 2. Materials and Methods

### 2.1. Selection and Eligibility Criteria

We used the PRISMA (Preferred Reporting Items for Systematic Reviews and Meta-Analyses) [20] guidelines to formulate the basis of pre-specified eligibility criteria using the PICO (P—Populations/People/Patient/Problem, I—Intervention(s), C—Comparison, O—Outcome) worksheet and search strategy (Table 1) [21].

Only prospective longitudinal studies (prospective cohort studies and randomized or non-randomized intervention trials) were eligible for inclusion; cross-sectional and retrospective studies being excluded. We included estimates based on assessment of outcome 12 months, or as close to 12 months as possible, after assessment of exposure. Articles analysing parental supply based on adolescent-, parent-, or both adolescent- and parent-report were eligible for inclusion. Only peer-reviewed journal articles published in English were included and there were no exclusion criteria regarding year of publication.

### 2.2. Search Strategy

Eight electronic databases were searched (Medline, MEDLINE In-Process and Other Non-Indexed Citations, EMBASE, PsycINFO, CINAHL, Scopus, Dissertations and Theses, and Cochrane Library) with the last search carried out on 10 September 2016. We searched for the following terms: parental provision, social hosting, parental source of alcohol, youth, student, teenage, underage, minor, risky drinking, excessive drinking, and binge drinking. We modified and used appropriate mesh terms in the databases with the assistance of the health librarian at the University of Newcastle. Table A1 presents an example of a search strategy performed in EMBASE where 107 articles were found. We screened titles and abstracts using the eligibility and exclusion criteria. Potential eligible articles for data extraction were identified after full-text review. Two reviewers (S.S. and M.K.) independently performed these two stages of screening. Disagreements were resolved by consensus or after consultation with a third reviewer (K.K.). Forward (Google Scholar) and backward searches (bibliographies of included articles) were conducted to find articles that might have been missed during initial database searches. A third reviewer (K.K.) independently reviewed the final included articles to confirm they met the inclusion criteria. The review was registered in PROSPERO [22] on 21 January 2016, prior to the analysis being undertaken (registration number CRD42016032409).

### 2.3. Data Extraction and Validity Assessment

Two authors (S.S. and M.K.) extracted information (population, intervention, outcome, study design, statistical methods, and results) using the Cochrane Public Health Group Data Extraction and Assessment Template [23] to tabulate findings of included articles. Finally, they independently assessed risk of bias using the Newcastle–Ottawa Scale for evaluating the quality of nonrandomized studies in meta-analyses [24]. Three factors were considered to score the quality of included studies: (1) selection, including representativeness of the exposed cohort, selection of the non-exposed cohort, ascertainment of exposure, and demonstration that at the start of the study the outcome of interest was not present; (2) comparability, assessed on the basis of study design and analysis, and whether any confounding variables were adjusted for; and (3) outcome, based on the follow-up period and cohort retention, and ascertained by independent blind assessment, record linkage, or self-report. We rated the quality of the studies (good, fair and poor) by awarding stars in each domain following the guidelines of the Newcastle–Ottawa Scale. A “good” quality score required 3 or 4 stars in selection, 1 or 2 stars in comparability, and 2 or 3 stars in outcomes. A “fair” quality score required 2 stars in selection, 1 or 2 stars in comparability, and 2 or 3 stars in outcomes. A “poor” quality score reflected 0 or 1 star(s) in selection, or 0 stars in comparability, or 0 or 1 star(s) in outcomes (Table 1).

### 2.4. Statistical Analysis (Meta-Analysis)

For articles that reported suitable statistics, a meta-analysis with a random effects model was conducted [25], using the *metan* command, specifying *random*, in Stata 13 [26]. There was methodological heterogeneity, studies having applied different measures of exposure and outcome. One study [27] reported results as correlation coefficients. For meta-analysis, we transformed the correlation coefficients into standardized mean differences and then converted them into log odds ratios (logORs) and standard errors (SElogORs). For binary outcomes, ORs and SEs were transformed into logORs and SElogORs. Finally, we pooled logORs and SElogORs of each study to produce summary effect sizes in a forest plot as an OR with 95% confidence intervals (CI). Heterogeneity of findings was assessed using χ^2^ and *I*^2^ statistics [28]. Analyses with *p* < 0.05 were interpreted as significant. We conducted a sensitivity analysis by examining change in the overall estimate after removing each study in turn, excluding the weaker studies, and excluding those studies that assessed both parent and child report. We assessed publication bias using funnel plots, contour-enhanced funnel plots, and both Begg’s [29] and Egger’s [30] tests.

## 3. Results 

**Figure 1:**
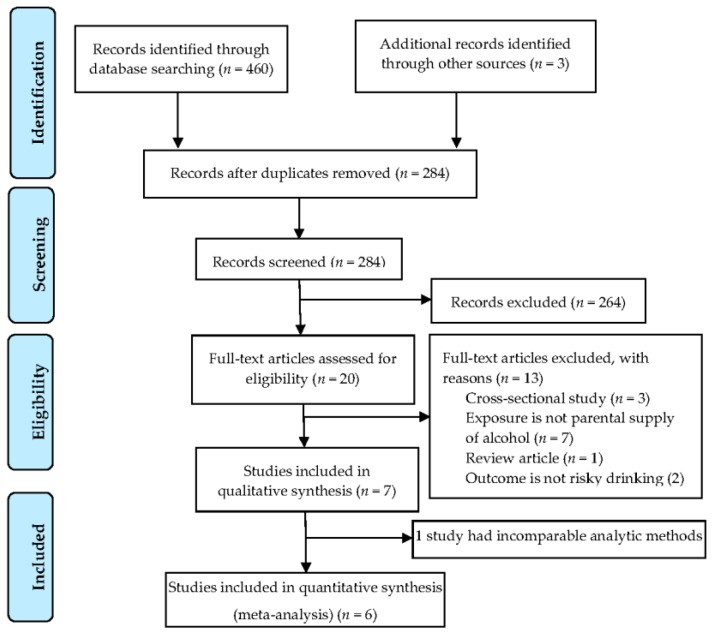
PRISMA (Preferred Reporting Items for Systematic Reviews and Meta-Analyses) study flow diagram.

Figure 1 summarises the selection of articles for review. Initial database searches identified 460 records and these were imported into Endnote X7 [31]. From backward and forward searches, three additional articles were identified for further screening. After removing 168 duplicate articles, 284 remained for title and abstract screening. Articles that did not meet inclusion criteria were not carried forward for full-text review, i.e., review articles, conference abstracts, cross-sectional or retrospective studies, studies in which the exposure was not parental supply of alcohol, or where the outcome was not risky drinking. Twenty full-text articles were assessed closely for eligibility, resulting in seven eligible articles from which data were extracted, and results summarised. Of these seven studies, suitable summary statistics were available from six studies for meta-analysis. The remaining one article [32] used analytic methods that do not produce effect estimates that can be converted to ORs.

### 3.1. Study Characteristics

Two studies were conducted in Sweden, two in the USA, one in The Netherlands, one in Australia and one each in the USA and Australia (Table 2). The follow-up period for all studies was ≥12 months, and samples ranged in age from 12 to 15 years at baseline. The age at last follow-up ranged from 14 to 31 years. Sample sizes ranged from 428 to 1945 participants. Parental supply of alcohol was reported by an adolescent, or by both a parent and an adolescent. Most studies were conducted in school settings, and all were published during 2003–2015.

Parental supply of alcohol was defined in different ways across studies, including alcohol being supplied at home, direct offers of alcohol by parents to their children in different drinking contexts (home alone, in a party, pub or club, in a park or car), and alcohol consumption at home on weekdays versus weekends. Outcomes included a range of definitions such as heavy episodic drinking (≥5 drinks on a single occasion) [33], problem drinking (as per the Rutgers Alcohol Problem Index [34], or lifetime DSM-IV alcohol abuse and dependence, based on 16- and 14-item scales [34], respectively), drunkenness (fell down or became sick due to alcohol use) [35], risky drinking (≥5 drinks on a single occasion) [36], and alcohol-related harm [27,37].

### 3.2. Summary of Study Findings

The study results are summarised in Table 2. In all of the studies, parental supply of alcohol was associated with increased risky drinking in mid- or late adolescence. In one study, the association was not significant for boys; however, the point estimate was in the hypothesised direction [33]. Table 3 provides quality scores for the studies, assessing risk of bias. Three studies were of good quality [27,33,36], one was of fair quality [38], and three were of poor quality [32,35,37]. A causal inference is constrained by risk of bias in some studies, the main concerns being measurement of the exposure (a lack of distinction between sips and whole drinks) [27,32,33,36,37], the lack of adjustment for key potential confounders (e.g., parent drinking, and parent rules about alcohol) [27,36,37,38], or a lack of clarity as to whether key confounders had been adjusted for [32,33,35]. 

#### 3.2.1. Drinking at Home or with Family

Two studies focused explicitly on drinking at home as an exposure. In a study of Australian adolescents (wave 1, mean age 15 years), Degenhardt and colleagues found that drinking at home with family in mid-adolescence was associated with a higher risk of drinking in a range of unsupervised settings, and of becoming a risky drinker in late adolescence [36]. In a Dutch study, Van der Vorst et al. found that drinking at home in early adolescence was associated with problem drinking later in adolescence, the association being similar irrespective of whether the drinking occurred with parents or peers [32]. 

In a USA cohort, Warner and White [37] found that an onset of drinking before age 11 years in family gatherings was associated with increased risk of “problem drinking” between 3 and 19 years later (OR = 2.86, 95% CI = 1.36, 6.00). Early onset of drinking outside family gatherings was associated with substantially higher risk of later problem drinking (OR = 8.32, 95% CI = 2.28, 30.4) [37].

#### 3.2.2. Drinking under Adult Supervision 

In a comparison of cohorts in the USA state of Washington, and the Australian state of Victoria, alcohol use among 14 year-olds under adult supervision either “at parties” or “at dinner or a special occasion” was found to be associated with higher levels of alcohol-related harm a year later (correlation coefficient = 0.22, *p* < 0.05) [27]. 

#### 3.2.3. Parental Supply and Offers of Alcohol

In a study of USA children, parental supply of alcohol at age 12 years was associated with an increasing trajectory of drunkenness and risky drinking [38]. In a Swedish cohort, parental supply of alcohol at home was associated with increased lifetime prevalence of drunkenness in boys (OR = 1.95, 95% CI = 1.18, 3.20) and girls (OR = 2.76, 95% CI = 1.54, 4.95) compared with adolescents who were not supplied with alcohol [35]. In another Swedish cohort, parental offers of alcohol to 7th graders (aged 13 years) were associated with increased risky drinking in 9th grade (aged 15 years). In adjusted models the association was significant for girls (OR = 1.80, 95% CI = 1.18, 2.75) but not for boys (OR = 1.25, 95% CI = 0.83, 1.89) [33]. 

### 3.3. Assessment of Study Validity

#### 3.3.1. Selection

Our search revealed a small number of prospective cohort studies from four high income countries with traditionally restrictive approaches to alcohol [39]. It is plausible that the association between parental supply and adolescent risky drinking is different in countries in which drinking small amounts more frequently is the prevailing consumption pattern, e.g., those in southern Europe [40]. Non-exposed groups were selected from the same source population as the exposed group in all studies.

#### 3.3.2. Measurement of Exposure and Outcome 

The exposures of interest were ascertained from child report in four studies [27,33,36,38] and from reports of both a parent and the child in the other three studies [32,35,37]. If participants generally under-reported parental supply (non-differential misclassification), ORs would be attenuated [41], i.e., the true increase in risk of adolescent risky drinking associated with parental supply would be larger than the estimates suggest. 

There is evidence to suggest that parents are not a reliable source of information about whether they supply their children with alcohol. In a study involving an anonymous survey of New Zealand school children aged 13–17 years, and a telephone (confidential but not anonymous) survey of their parents, 36% of children reported that their parents had given them alcohol to drink in unsupervised settings in the preceding month, while only 2% of parents reported that they had supplied alcohol to their children for unsupervised drinking in the same period [42]. It is unknown whether such misreporting would be differential or non-differential with respect to the outcome of adolescent risky drinking, such that the likely direction of bias in the estimate of association is indeterminable. This uncertainty about the effects of misclassification of exposure also applies to the problem of counting sips as drinks.

The effects on estimates of systematic misreporting of the outcome are also difficult to assess and depend on whether misreporting varies as a function of exposure status [41]. Methodological research suggests that reporting of alcohol consumption is fairly robust in conditions in which respondents have no reason to expect judgement (negative or positive) from researchers, parents, or peers, on the basis of their responses, e.g., where questionnaires are completed anonymously [43].

Studies involved parental consent [27,32,33,35,36,37,38,44] and student assent [27,37,38] prior to data collection. In three studies the paper specifically indicated that participants were assured of confidentiality [33,35,38], and in two it was noted that participants were given the opportunity to refuse to participate or answer questions [35,38]. It is unclear what conditions prevailed in the other studies, though it should be noted that in all of the papers it was stated that ethical approval had been received from an institutional review committee.

#### 3.3.3. Confounding

Several studies [27,32,36,37,38] either did not use multivariable analyses to model outcomes [27,32,36] or did not clearly specify what potential confounders were adjusted for [32,35]. Likely confounders include parental drinking, peer and sibling drinking, family income, ethnicity, and religiosity, all of which have been found in prospective cohort studies to be associated with the outcome, namely adolescent risky drinking (e.g., [45]), and are plausibly associated with the exposure (parental supply) [46]. Accordingly, it is likely that effect estimates have been inflated by confounding.

#### 3.3.4. Attrition

Rates of loss-to-follow-up ranged from 3% to 15%, suggesting a low overall potential for attrition bias. The median duration of follow-up was ≥12 months, a period probably long enough for outcomes to occur if parental supply were a causal factor.

### 3.4. Meta-Analysis

**Figure 2:**
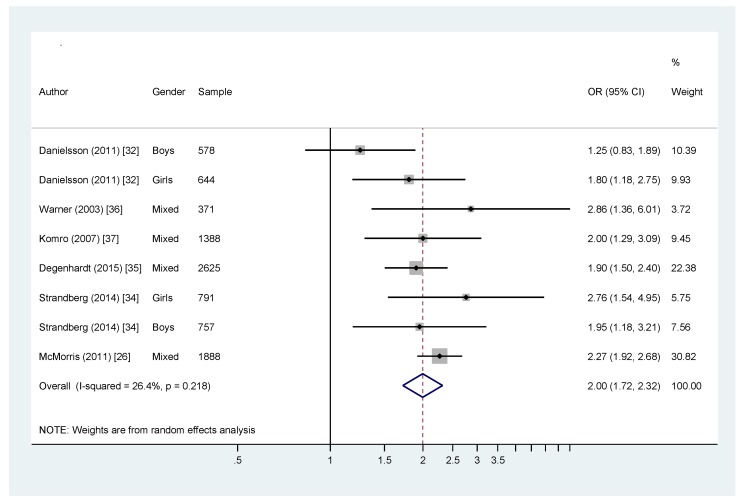
Meta-analysis forest plot.

Of the six studies with data suitable for meta-analysis, two estimated ORs stratified by sex, while the remaining four reported combined ORs, producing a total of eight estimates. Figure 2 presents a forest plot with effect sizes and 95% CIs. All of the ORs were >1, indicating that parental supply of alcohol was associated with twice the odds of later adolescent risky drinking (OR = 2.00, 95% CI = 1.72, 2.32; *I*^2^ = 26.4%; *p* = 0.218). The *I*^2^ statistic indicates that the estimates are consistent across the studies. We found the effect estimates from sensitivity analyses were consistent with the effect estimate from the primary analysis (Table A2).

One study [32] used analytic methods (path analysis) producing estimates of association that we could not include in the meta-analysis. It found positive associations between parental supply of alcohol and adolescent risky drinking, making it at least broadly consistent with the meta-analytic results.

#### Publication Bias

**Figure 3:**
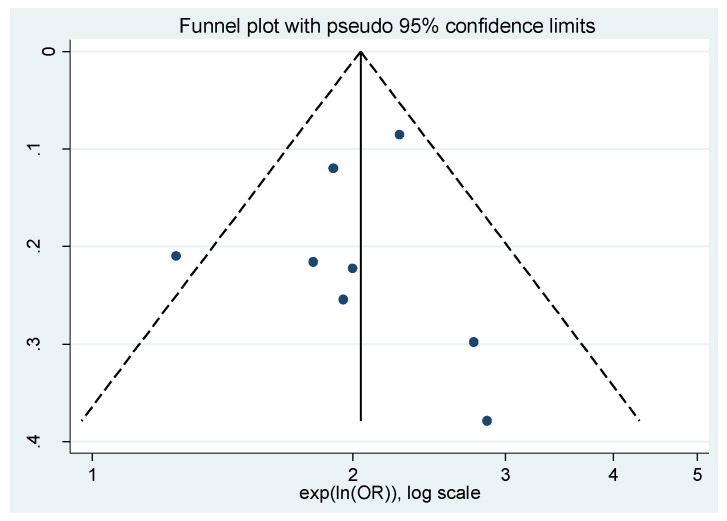
Funnel plot of the eight estimates available for meta-analysis. SE: Standard error.

**Figure 4:**
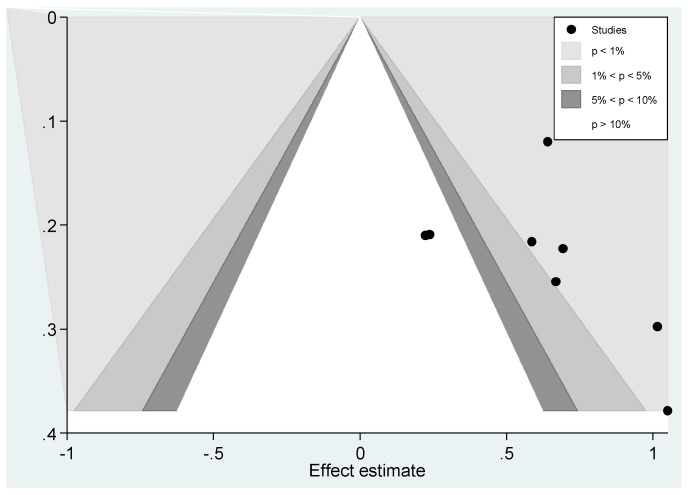
Contour-enhanced funnel plot of the eight estimates available for meta-analysis.

The funnel plot (Figure 3) is asymmetrical, suggesting the possibility of publication bias. A contour-enhanced funnel plot (Figure 4) also suggests the possibility of publication bias as missing studies are mostly in the non-significance area. Begg’s (*p* = 0.336) and Egger’s test results (*p* = 0.689) do not confirm this observation; however, they are limited by the small number of studies.

## 4. Discussion

The results of this systematic review and meta-analysis suggest the possibility that parental supply of alcohol in childhood increases the odds of later adolescent risky drinking; however, a causal inference is limited by a high likelihood that estimates are inflated by a lack of control for confounders, and a risk of publication bias. If the findings do reflect a true effect, the following aspects of parental supply are implicated: direct supply of alcohol by parents, offers of alcohol by parents, adolescent drinking under parental supervision, adolescent drinking at home, and adolescent drinking in family gatherings.

We included only prospective cohort studies providing the basis for establishing that parental supply preceded the outcome of adolescent risky drinking, excluding simple reverse causality as an explanation for the association. However, we cannot exclude more complex competing explanations for the findings. For example, parental supply may initially facilitate moderate drinking in early or mid-adolescence which in turn potentiates heavier drinking and thereby demands on parents or peers to supply alcohol in the larger amounts necessary for risky drinking. Testing such explanations requires assessment at multiple time points and analytic approaches (e.g., marginal structural models [47]) that can model iterative (i.e., time-dependent) processes.

Strengths of the review include the comprehensive search strategy, independent screening, study identification and coding, and the risk of bias assessment. The use of meta-analysis increased the precision of the key point estimate, and formal assessment of publication bias has helped to qualify that estimate. Some studies were judged to be high in risk of bias, particular concerns being unreliable measurement of exposure, and lack of adjustment for confounding variables. It is possible that the literature is biased by non-publication of small studies with null findings or findings suggesting that parental supply is protective against adolescent risky drinking.

### Limitations

We standardized effect estimates for the purpose of comparison. The transformations we performed (e.g., correlation coefficient to Cohen’s *d* to lnOR) may have introduced error producing wider confidence intervals for estimates. 

The variety in definition and measurement of exposures was sufficient to compromise the comparability of studies and it highlights the importance of context in the construct of parental supply. For instance, Warner and White [37] did not define what drinking in a family gathering meant in practice. We assumed it included parents supplying alcohol to their children to drink at family gatherings. Conversely, we assumed drinking outside family gatherings, e.g., with peers, did not involve parental supply, yet qualitative research suggests it is likely that in some situations more complex combinations of parent and peer supply occur [48]. 

In the study by Danielsson and colleagues [33], we deemed “parental offers of alcohol” as equivalent to “parental supply of alcohol”; however, the paper does not indicate whether adolescents accepted the offers. Contact with the authors confirmed that the questions asked did not permit a judgement to be made about whether the offers resulted in supply or consumption. We reasoned that, in any case, an offer alone may plausibly confer risk by communicating a permissive attitude toward adolescent drinking, as some survey data suggest [49]. Similarly, the exposure “adult supervised drinking” (used in [27]) does not define the relationship of the adult supervisor to the adolescent drinker, such that some instances of what were treated as parental supply may in fact have been supply by other adults.

Whether children were allowed to drink whole beverages or merely sip their parents’ alcoholic beverages under supervision was not distinguished in most studies [27,32,33,36,37,44]. In the wider literature on drinking initiation, sipping is often categorized as drinking, yet there is evidence from one prospective cohort study that, in contrast to consuming whole drinks, sipping is not associated with later risky drinking [50]. 

In a recently published prospective cohort study we found that parental supply of alcohol (of whole drinks, not merely sips) measured when children were around 13 years-old, was not associated with risky drinking (>4 drinks in a single episode in the preceding year) up to three years later, after adjustment for parental drinking, access to alcohol without parents knowing, alcohol-specific rules, monitoring, family factors, family alcohol problems, child factors, and peer factors [51]. Importantly, unadjusted analyses showed a positive association between parental supply of alcohol and risky drinking that disappeared in multivariable models. The study had high retention (>85% three years after baseline), and the cohort is broadly representative of the Australian population of the same age [52], however, it remains possible that evidence of risk associated with parental supply will emerge as members of this cohort enter their late teens, when the prevalence of risky drinking typically increases sharply in Australia.

## 5. Conclusions

Prospective cohort studies suggest that parental supply of alcohol in childhood increases the likelihood of risky drinking later in adolescence but the potential for bias in this literature is judged to be high. Further longitudinal studies are needed, with particular attention to distinguishing parental supply of sips versus whole drinks, the meaning of supervised drinking, measurement of likely confounders, and adjustment for them in multivariable models. Studies are needed in cultures with traditionally low restrictions on youth drinking (so-called “wet” societies, e.g., in southern Europe [40]), and in low and middle income countries where alcohol consumption is increasing as economies grow rapidly [53].

## Figures and Tables

**Table 1:** PICO Worksheet (parental supply of alcohol and adolescent risky drinking).

**Population**	Adolescents who could have been exposed to parental supply of alcohol prior to the age of 18.
**Intervention**	Parental supply of alcohol
**Comparison**	Children who were exposed versus unexposed to parental supply of alcohol
**Outcome**	Adolescent risky drinking is defined as consumption of ≥5 drinks on a single occasion, at least monthly. A range of terms was used in the literature in reference to consumption at the risk level we defined, or at higher risk: alcohol misuse, drunkenness, alcohol dependence, heavy drinking, binge drinking, intoxication, heavy episodic drinking. We considered these as falling within the category “risky drinking”.

PICO: Population, Intervention, Comparison, Outcome.

**Table 2:** Study characteristics and results.

Author	Study Design	Location	Follow-Up Period (Years)	Sample (*n*)	Age at Baseline (Years)	Exposure	Outcome	Statistical Method	Results
Danielsson et al. (2011) [33]	Prospective study	Sweden	2	1222 adolescents	13	Parents’ offer of alcohol	Risky drinking ^1^	Simple and multivariable logistic regression	Parental offer of alcohol increased the risk for HED in the ninth grade for girls (OR = 1.8, 95% CI = 1.2, 2.8) only.
Degenhardt et al. (2015) [36]	Prospective study	Australia	2	2625 adolescents	14.9	Drinking at home with family	Risky drinking (past week) ^2^	Repeated measures discrete time proportional hazards models	Adolescents reported that those who drank with family more than 3 times were more likely to drink riskily in later adolescence (RR = 1.9, 95% CI = 1.5, 2.4).
Komro et al. (2007) [38]	Prospective study	USA	2	1388 adolescents 1388 parents	12	Received alcohol from parents.	Drunkenness Risky drinking (past 2 weeks) ^2^	Generalized linear mixed-model regression	A significant increase in the trajectory of drunkenness (OR = 2.3, 95% CI = 1.5, 3.4) and HED (OR = 2.0, 95% CI = 1.3–3.2) was observed when students, at age 12, reported that at the last time they drank they received alcohol from their parent.
McMorris et al. (2011) [27]	Prospective study	USA (Washington State) and Australia (Victoria);	1	1888 adolescents 1888 parents	13	Adult supervised alcohol use	Alcohol-related harm	Two-group multiple-group path models	In both states, adult-supervised alcohol use among 8th grade students was associated to later alcohol use and alcohol related harms in 9th grade (correlation coefficient = 0.22).
Strandberg et al. (2014) [35]	Prospective study	Sweden	2.5	1752 adolescents 1314 parents	13	Alcohol servings to youth at home.	Drunkenness (past month)	Multilevel logistic regression	Adolescents who were being supplied alcohol at home in the 7th grade were more likely have ever been drunk in the 9th grade compared to non-supplied adolescents. Girls: OR = 2.8, 95% CI = 1.5, 5.0 Boys: OR = 2.0, 95% CI = 1.2, 3.2 Supply of alcohol at home did not significantly predict frequent drunkenness in adolescents. Girls: OR = 1.26, 95% CI = 0.74, 2.15 Boys: OR = 1.24, 95% CI = 0.80, 1.92
Van der Vorst et al. (2010) [32]	Prospective study	Netherlands	3	428 ^3^	15.22 (older sibling) 13.36 (younger sibling)	Drinking alcohol at home.	Problem drinking	Structural path analysis	For both older and younger siblings, drinking alcohol at home and outside home in mid-adolescence predicted problem drinking in late adolescence. $\chi_{older}^{2}$(1) = 0.34, *p* > 0.05; $\chi_{younger}^{2}$(1) = 0.20, *p* > 0.05.
Warner & White (2003) [37]	Prospective study	USA	3, 6, 13, and 18	371 adolescents	12	Drinking alcohol at a family gathering.	Alcohol use-related problems	Hierarchical logistic regression models	Participants who had their first drink at a family gathering before the age of 11 are significantly more likely to develop problems associated with alcohol use compared to participants who were more than 11 years old (ORs = 2.9, 95% CI = 1.4, 6.0).

^1^ Frequency of drinking six cans of medium-strength beer or four cans of normal beer or four large bottles of strong cider, or a bottle of wine, or half a bottle of spirits on an occasion; ^2^ Drinking ≥5 drinks in a row; ^3^ Families (father, mother, and two siblings). CI: Confidence interval; HED: Heavy episodic drinking; OR: Odds ratio; RR: Relative risk.

**Table 3:** Risk of bias assessment (Newcastle–Ottawa Quality Assessment Scale criteria).

Study	Selection				Comparability	Outcome			Quality Score
	Representativeness of Exposed Cohort	Selection of the Non-Exposed Cohort from Same Source as Exposed Cohort	Ascertainment of Exposure	Outcome of Interest Was Not Present at Start of Study	Comparability of Cohorts	Assessment of Outcome	Follow-Up Long Enough for Outcome to Occur (Median Duration of Follow-Up ≥6 Months)	Adequacy of Follow-Up	
Danielsson et al. (2011) [33]	Participants were truly representative of adolescents of Stockholm, Sweden. Participants covered low, middle and high socio-demographic profiles and participated from 6 districts (18 schools and 79 classes) of Stockholm out of 18 districts. ★	Yes ★	Students answered questionnaires in school	Yes ★	Early alcohol debut (proportion of friends who drink, smoking, truancy, bullying, more than 300SEK to spend per month), protective factors (more than 6 h spent with parents on weekends, relationship to parents and peers), parental monitoring, school environment were adjusted for multivariable logistic regression. ★	Adolescent self-report	Yes ★	87% of adolescents participated at the first data collection and after two years 85% participated at the second data collection. ★	Good
Degenhardt et al. (2015) [36]	Adolescents were truly representative of the community. Schools were randomly selected from a stratified frame of government, independent private and Catholic schools. From each type of school the probability of selection was proportional to the number of students of that age. ★	Yes ★	Students completed questionnaires by computer at school	Yes ★	Wave of observation, sex, school location, parental separation/divorce, frequency of parental drinking, smoking, adolescents’ smoking, cannabis use, antisocial behaviour and signs of anxiety and depression were adjusted for repeated measures discrete time proportional hazards models. ★★	Adolescent self-report	Yes ★	87% participated at the 6-month follow-up, 84% at the 12-month follow-up, 81% at the 18-month follow-up and 79% at 24-month follow-up. ★	Good
Komro et al. (2007) [38]	Participants were not representative of adolescents of Chicago, USA. Only Chicago public schools were selected and students were predominantly African American (44%) or Hispanic (39%) and low income (79%).	Yes ★	Parents completed survey at home and students completed at school	Yes ★	Race/ethnicity, age, gender and family composition, parent/child communication, family alcohol discussions, peer alcohol use, peers’ supply of alcohol, parental monitoring and alcohol communication were adjusted for generalized linear mixed-model regression. ★★	Adolescent self-report	Yes ★	Between 91% and 96% participated at each of the 12-month and 24-month follow-up. ★	Fair
McMorris et al. (2011) [27]	Representative samples were recruited from seventh grade students of Victoria and Washington states of Australia and USA respectively. ★	Yes ★	Students completed questionnaires at classroom	Yes ★	Gender, age, and socioeconomic status were adjusted for path models. ★	Adolescent self-report	Yes ★	97% participated at 12-month follow-up and 24-month follow-up. ★	Good
Strandberg et al. (2014) [35]	40 municipal schools participated from 13 counties out of 21 Swedish counties. ★	Yes ★	Parents received questionnaires by post and youth completed questionnaires in school	Yes ★	Multilevel logistic regression. What confounders were adjusted for was not clearly stated.	Adolescent and parent self-report	Yes ★	92% adolescents and 75% parents participated at the 12-month follow-up and 88% adolescents and 68% parents participated at the 30-month follow-up.	Poor
van der Vorst et al. (2010) [32]	Participants were representative of two biological parent households of 20 municipalities of Netherlands. There were inclusion criteria of participants that indicate “parents had to be married or living together, and the siblings and their parents had to be biologically related”. ★	Yes ★	Family members (both parents and two adolescent children) completed questionnaires at home in the presence of a trained interviewer	Yes ★	Structural path analysis. What confounders were adjusted for was not clearly stated.	Adolescent self-report	Yes ★	416 families participated at the 12-month follow-up and 404 families participated at the 24-month follow-up. ★	Poor
Warner & White (2003) [37]	Participants were representative of white adolescents (89%) who lived in metropolitan, middle-class and working environment.	Yes ★	Parents and adolescents completed self-reported questionnaires at home during recruitment and later completed in the project site	Yes ★	Gender, socioeconomic status, religion were adjusted for hierarchical logistic regression models. ★	Adolescent and parent self-report	Yes ★	91% participated at 3-year follow-up, 6-year follow-up and 13-year follow-up. Participation rate is not specified at the 18-year follow-up.	Poor

Good quality: 3 or 4 stars (★) in selection domain AND 1 or 2 stars in comparability domain AND 2 or 3 stars in outcome domain; Fair quality: 2 stars in selection domain AND 1 or 2 stars in comparability domain AND 2 or 3 stars in outcome/exposure domain; Poor quality: 0 or 1 star in selection domain OR 0 stars in comparability domain OR 0 or 1 stars in outcome/exposure domain.

## Supplementary Materials

The following are available online at www.mdpi.com/1660-4601/14/3/287/s1, Table S1: PRISMA Checklist.

## Appendix A.

**Table A1:** Search strategy used for EMBASE.

A. Parent Rules	B. Adolescent	C. Risky Drinking	D. Study Design
1. ((parent* or mother* or father* or maternal* or guardian* or custodian*) adj5 (provision* or approv* or suppl* or influence* or permissive* or host* or offer or furnish or source* or allow* or permission* or permit or agree*)).mp. (33999)	2. child*.mp. (2213989) 3. offspring.mp. (60119) 4. adolescen*.mp. (1373825) 5. famil*.mp. (1109647) 6. juvenil*.mp. (110083) 7. girl*.mp. (149109) 8. boy*.mp. (157168) 9. youth.mp. (48965) 10. pubescen*.mp. (2207) 11. teen*.mp. (30193) 12. young women.mp. (22130) 13. young men.mp. (13253) 14. “young male*”.mp. (12873) 15. “young female*”.mp. (8448) 16. student*.mp.(332349) 17. young people.mp. (24628) 18. minor*.mp. (287800) 19. kid*.mp. (1036340) 20. underage*.mp. (1013) 21. puber*.mp. (47802) 22. early adult.mp. (1242) 23. young adult*.mp. (167841) 24. exp high school/ (11830) 25. exp progeny/ (34734) 26. 2 or 3 or 4 or 5 or 6 or 7 or 8 or 9 or 10 or 11 or 12 or 13 or 14 or 15 or 16 or 17 or 18 or 19 or 20 or 21 or 22 or 23 or 24 or 25 (5322013)	27. exp alcoholic beverage/(21773) 28. exp alcohol intoxication/(11605) 29.liquor.mp. (8808) 30. Heavy drink*.mp. (7354) 31. exp alcoholism/(106005) 32. exp binge drinking/(1831) 33. heavy episodic drink*.mp. (630) 34. problem drink*.mp. (3129) 35. excessive drink*.mp. (1177) 36. risky drink*.mp. (646) 37. hazardous drink*.mp. (1005) 38. 27 or 28 or 29 or 30 or 31 or 32 or 33 or 34 or 35 or 36 or 37 (149053) 39.26 and 38 (35524)	40. longitudinal stud*.mp.(106835) 41. cohort stud*.mp. (143528) 42. prospective study.mp. or prospective study/(355640) 43. retrospective study.mp. (448846) 44. 40 or 41 or 42 or 43 (975260) 45. randomized controlled trial/(392091) 46. 44 or 45 (1376262) 47. random*.tw (1053065) 48. Clinical Trials/(68194) 49. 46or 47 or 48 (2233152)
50. A (1) and B (39) and C (49) = 107			

**Table A2:** Sensitivity analysis.

Studies for Sensitivity Analysis	No. of Studies (No. of Estimates)	OR	95% CI (Lower, Upper Limit)	*p* for Heterogeneity ($I^{2}$(%))
All except Danielsson (2011) (boys)	6 (7)	2.12	1.89, 2.39	0.730 (0)
All except Danielsson (2011) (girls)	6 (7)	2.01	1.70, 2.38	0.166 (34.3)
All except Warner (2003)	5 (7)	1.96	1.68, 2.29	0.191 (31.0)
All except Komro (2007)	5 (7)	1.99	1.67, 2.36	0.147 (36.8)
All except Degenhardt (2015)	5 (7)	2.01	1.66, 2.43	0.171 (33.6)
All except Strandberg (2014) (girls)	6 (7)	1.96	1.68, 2.28	0.071 (46.3)
All except Strandberg (2014) (boys)	6 (7)	1.99	1.68, 2.36	0.149 (36.7)
All except McMorris (2011)	5 (7)	1.88	1.59, 2.22	0.338 (12)
Excluding studies rated as poor quality	4 (5)	1.90	1.57, 2.29	0.109 (47.1)
Excluding studies that assessed both parent and child self-report	4 (5)	1.90	1.57, 2.29	0.109 (47.1)

No.: Number.
